# To be assertive or not to be assertive: That is the question! Students' reactions to sexual harassment in academia

**DOI:** 10.3389/fpsyg.2022.949103

**Published:** 2022-09-20

**Authors:** Cristina Cabras, Cristina Sechi, Mirian Agus, Ester Cois, Clementina Casula, Luigi Raffo, Oriana Mosca

**Affiliations:** ^1^Department of Pedagogy, Psychology, Philosophy, University of Cagliari, Cagliari, Italy; ^2^Department of Political and Social Sciences, University of Cagliari, Cagliari, Italy; ^3^Department of Literature, Languages and Cultural, University of Cagliari, Cagliari, Italy; ^4^Department of Electrical and Electronic Engineering, University of Cagliari, Cagliari, Italy

**Keywords:** sexual harassment reactions, university students, sex, gender roles, academia

## Abstract

**Introduction:**

In the literature, no integrated definition of sexual harassment (SH) occurs but there is clear unanimity about SH being offensive, humiliating, and intimidating behavior. Within academic settings, SH has severe negative effects on students' physical or emotional wellbeing as well as on their ability to succeed academically.

**Methods:**

The aim of this study was to investigate the relationship between sex, gender roles, and the ways to manage SH (assertive and nonassertive reactions) in university students. It was hypothesized that female students would report more nonassertive reactions compared to male students. In addition, following the Bem theory on gender roles and using the self-report tool by the same author, it is hypothesized that female and male students, who are classified as feminine, will report more nonassertive responses, whereas male and female students, who are classified as masculine, will report more assertive responses. Our hypothesis was tested with a sample of 1,415 university students (593 men, 41.9%, and 822 women, 58.1%) who completed a questionnaire approved by the local ethical review board for research from the end of January 2019 to the first half of February 2019.

**Results:**

Contrary to our hypothesis, results showed that women react more than men in both assertive and nonassertive modalities. In addition, our results confirmed the main effect of both sex and gender roles on students' assertive and nonassertive reactions to SH in academia.

**Conclusion:**

Educational programs about SH may prove useful in preventing its occurrence. Gender equality plans in academia can improve a nonsexist and safe environment for students. It is urgent to improve transparency and accountability of policies on the management of SH: academic institutions need to formulate a procedure to facilitate SH reporting, considering the sensitive balance of confidentiality and transparency issues. Support for the victims (social services, healthcare, legal representation, and advice concerning career/professional development) must be included.

## Introduction

Sexual harassment (SH) may take many various forms from less explicit (e.g., verbal comments) to explicit forms (e.g., sexual abuse) (Fitzgerald et al., 1988; Gruber, 1992).

There is still a controversy on the definitions of SH and there is a lack of a definition that can be broad enough to understand the various characteristics and forms of the issue (Leitich, 1999).

Although no integrated definition of SH occurs (Hulin et al., 1996; Pina et al., 2009; Stockdale et al., 2014; Sabbag et al., 2018), there is clear unanimity about harassment being offensive, humiliating, and intimidating behavior which usually comprises the abuse of power given by the gender order of society and organizations (Cairns, 1997; Nicolson, 1997; Magley et al., 1999; Huerta et al., 2006; Cabras et al., 2018, 2022). Similarly, a behavior can be regarded as SH if it is undesirable or without the free approval of the victims. All the definitions agree on one fundamental issue that SH is, first of all, sexual in nature, illegal, unwanted, unwelcomed, and immoral behavior which determines serious outcomes for the victims.

In higher educational institutions, the issue of SH is gaining scientific attention and progressively becoming the focal point of academic debate (Taiwo et al., 2014) because it often denies or limits a student's ability to participate in or benefit from a university's education program (Karami et al., 2020). Within academic settings, SH has severe negative effects on students' physical or emotional wellbeing as well as on their ability to succeed academically (Hill and Silva, 2005; Huerta et al., 2006; Willness et al., 2007). A survey showed that approximately 12% of the students across 27 universities experience some form of nonconsensual sexual contact by physical force (Cantor et al., 2015). In the European context, a study revealed that 77% of women students experienced some form of SH in their academic life (Fasting et al., 2014). Different authors have shown that 59% of US women have experienced SH, and women with higher education are far more likely to say they have experienced harassment compared to less educated women (Karami et al., 2020). In a report by the US National Academies of Science, Engineering, and Medicine survey of academic environments, 50% of female faculty/staff and 20–50% of female students reported SH experiences (National Academies of Sciences, 2018). This report described different negative professional outcomes for both faculty and staff (e.g., important declines in work satisfaction and engagement and productivity) and students (e.g., dropping courses and classes or receiving lower grades) (National Academies of Sciences, 2018). SH has a tremendous impact on mental health, such as the development of anxiety and depression (psychological distress) and post-traumatic stress disorder (PTSD) symptoms, low self-esteem, and panic disorder (Jussen et al., 2019). SH can manifest also with psychosomatic manifestations such as nausea, fatigue, frequent headache, sleep problems, respiratory infections, weight management issues, and gastrointestinal problems (Thakur and Paul, 2017). A large body of reliable data demonstrates that experiencing SH, even at low levels of frequency and intensity, can lead to psychological wellbeing worsening and increments in psychological distress, including major emotional disorders. Although not every individual who is exposed to such experiences will develop symptoms, such reactions are more common than not—indeed, they appear to be the normative response (Fitzgerald and Cortina, 2017). Despite various studies on the nature, reasons, and effects of SH in the university environment, much is not known about the different forms of reactions to which individuals choose to respond to SH. In particular, the study of students' responses to SH is relatively unexplored in the Italian university context.

The ways individuals manage SH differ according to the focus and the type of response (Gutek and Koss, 1993). The focus of the response can be categorized as either self-focused that does not comprise the harasser or harasser-focused that involves the harasser, while the type of response refers to the amount of outside support the victims seek, and it takes the form of either a self-response that is with no use of outside resources or a supported response that requires the use of outside resources (Gutek and Koss, 1993). Magley (2002) emphasized that there is a multiplicity of responses adopted by the victims in a dynamic process that unfolds over time (Fitzgerald and Cortina, 2017).

The individual's response to SH can be categorized either as assertive or passive (e.g., seeking social support or ignoring the behavior) or as nonassertive or active (e.g., reporting an incident to an authority) (Gruber and Bjorn, 1986; Cochran et al., 1997). Empirical support for this categorization is demonstrated by the studies of Cortina and Wasti (2005), which proposed a multilevel model of coping with SH, identifying three distinct patterns of coping (i.e., detached (nonassertive), avoidant negotiating (nonassertive), and support-seeking (assertive), each of which reflected relatively greater or lesser use of various combinations of behavior [see Knapp et al. (1997) for an earlier description of similar categories].

Usually, individuals who are more accepting of SH are less likely to consider their experiences as serious and, consequently, respond less assertively (Cochran et al., 1997). However, the person's choice of responses to SH might vary in many aspects, specifically regarding gender (Russell and Trigg, 2004).

It is plausible to hypothesize that women and men may use dissimilar strategies to respond to SH, particularly if the gravity and incidence of their experiences are different. Research suggests that women are more likely to ignore the harassment, avoid the harasser (Benson and Thomson, 1982; Gutek, 1985; Gutek and Koss, 1993; Cochran et al., 1997), or deflect the harassment by joking or going along with it (Gutek, 1985). In academic settings, some studies have shown that the majority of women who are victims of SH do not respond assertively either by directly confronting the harasser or reporting the harasser to a university institutional authority but instead respond rather passively to the harassment experience (e.g., abandoning the university place) (Rudman et al., 1995; Popoola, 2010; Arulogun, 2013).

As an additional gender-related aspect that may be linked to the responses of SH, it is important to examine gender roles. According to the schema theory proposed by Bem (1981), gender roles refer to behavioral systems and social roles that are seen as appropriate for women and men and include those essential emotions and feelings that are conventionally considered to represent what it means to be female or male.

Masculinity focuses on a variety of characteristics such as power, assertiveness, leadership, autonomy, and competitiveness. In contrast, femininity focuses on different characteristics such as empathy and emotional disposition that are typical aspects inverse to masculinity (Helgeson, 1994). These characteristics will lead masculine women and men to believe that they can control what happens to them and, therefore, face harassment through their acts or deliberately manage such behaviors (Russell and Trigg, 2004; Fischer, 2006). According to Bem's (1974), individuals who possess a high degree of both masculine and feminine traits are categorized as androgynous, which is the most adaptive gender feature. Moreover, individuals who manifest a decrement in both masculine and feminine traits are categorized as undifferentiated in this model. The relationship between gender roles and attitudes and reactions toward SH is not clear, as, in the literature, there are some equivocal results. Powell (1986) found that men with a high level of masculinity were less likely to view disturbing sexual remarks as SH compared to other men, while women with a high level of femininity were more likely to do so compared to other women; moreover, both men and women with high levels of femininity perceived slightly more actions as SH than did their counterparts. In the same direction, Russell and Trigg (2004) found that highly feminine men and women were less likely to tolerate SH compared to their less feminine peers. However, there have been some studies (Bursik, 1992; McCabe and Hardman, 2005; Bursik and Gefter, 2011) that did not find a relation between gender roles and perception and tolerance of SH. The purpose of this study was to explore sex and gender-role orientation differences as well as interaction effects between sex and gender-role orientation in response to an imagined sexual harassment.

It was hypothesized that female students would report more nonassertive reactions compared to male students (Rudman et al., 1995; Popoola, 2010; Arulogun, 2013). In addition, it is hypothesized that female and male students who are classified as feminine will report more nonassertive responses, whereas male and female students who are classified as masculine will report more assertive responses (Russell and Trigg, 2004; Fischer, 2006).

## Materials and methods

### Participants

Participants included 1,415 university students enrolled at the University of Cagliari (593 men, 41.9%, and 822 women, 58.1%) who completed a series of self-report measures approved by the local ethical review board for research. Participants' ages ranged from 18 to 67 years (M = 25.85; SD = 7.57).

### Procedure

The battery of self-report scales was administered to students at the University of Cagliari, attending heterogeneous courses from the end of January 2019 to the first half of February 2019. An email was sent to the whole population of students with an invitation to fill in the battery. The scales were administered using Lime Survey, an online survey tool, and took approximately 15 min to fill in. According to the ethical standards Declaration of Helsinki (World Medical Association, 2001), participants were informed about all relevant aspects of the study (e.g., methods and institutional affiliations of the researchers) before they started to fill in the questionnaire. Importantly, they were apprised of their right to anonymity, to refuse to participate in the study, or to withdraw their consent to participate at any time during the study without fear of reprisal. Participants then confirmed that they had understood the instructions correctly, agreed to participate, and began filling out the questionnaire.

#### Measures

##### BEM Sex Role Inventory

To assess the gender roles, participants filled in the Bem Sex Role Inventory (Bem's, 1974; Italian version De Leo et al., 1985). This self-report instrument is composed of 60 items, evaluating personality characteristics (20 referring to traditional feminine features, 20 referring to masculine features, and 20 referring to neutral features). Each feature was rated by a seven-point Likert scale (1 = never true to 7 = always true). The scores in femininity and masculinity were obtained by computing the mean of the 20 items belonging to each scale (femininity M = 4.62, SD = 0.769, median = 4.65, Cronbach's alpha = 0.836; masculinity M = 4.39, SD = 0.829, median = 4.40, Cronbach's alpha = 0.859). Then, the median split procedure was applied referring to the median of the sample for femininity and masculinity scales; this practice, identified in the literature, allows to find a typological variable, defined as *gender role*, having four modalities:

- feminine (under the median on masculinity and above the median on femininity);
- masculine (under the median on femininity and above the median on masculinity);
- androgynous (above the median on femininity and masculinity);
- undifferentiated (under the median on femininity and masculinity).

#### Reactions to sexual harassment in academia questionnaire (RSHAQ)

We created a theoretically based questionnaire (refer to Appendix 1 in Supplementary material) to evaluate individuals' imagined responses to sexual harassment by adapting items used by other researchers (Matsui et al., 1995). Specifically, these items were intended to evaluate individuals' imagined reactions to sexual harassment ranging from nonassertive to assertive.

*Nonassertive reactions*, which focused on changing one's own behavior to modify the situation, were (a) speak about the abuse with family and/or friends, (b) speak about the abuse with colleagues, and (c) avoid the intimate situations with the abuser.

*Assertive reactions*, which focused on modifying the behaviors of the actor, were (a) break the silence about the hypothesized abuse (inverse of silence), (b) denounce the abuser to university organizations, (c) denounce the abuser to the police, and (d) report the abuse to university professors.

Participants were asked to respond to each item on a five-point Likert scale ranging from 1 (never) to 5 (always).

### Statistical analyses

We calculated descriptive statistics and zero-order correlations, which are reported in Table 1.

**Table 1:** Means, standard deviations, skewness, kurtosis, and zero-order correlations between variables (*N* = 1,415).

	**Mean**	**SD**	**SK**	**C**	**1**	**2**	**3**	**4**	**5**	**6**
1. BEM_Masculine	4.392	0.818	−0.187	−0.037	1					
2. BEM_Feminine	4.621	0.758	−0.562	1.234	0.253**	1				
3. BEM_Neutral	4.275	0.552	−0.541	3.775	0.448**	0.654**	1			
4. Assertive Reactions	2.604	0.915	−0.115	−0.956	0.043	0.110**	0.070**	1		
5. Non-assertive Reactions	3.744	1.055	−0.913	0.337	−0.025	0.173**	0.061*	0.520**	1	

Notes. ** *p* < 0.01; * *p* < 0.05.

To assess the degree to which the items used to assess the reactions to harassment they were intended to measure, Principal Components Analyses (PCA) with Promax rotation was used as the extraction method for all the analyses conducted on the 7 items. Scree plots were used to determine the number of factors that would be examined. In addition, the items in each factor also had to conform to our theoretical expectations for the factor to be included as a subscale in the analyses.

Finally, we conducted a 2 × 4 MANOVA with two between-subjects factors, namely, (1) sex measured on two levels (male/female) and (2) gender roles measured on four levels (feminine, masculine, androgynous, and undifferentiated) on the two dependent variables, including nonassertive and assertive reactions.

## Results

Descriptive statistics and zero-order correlations are reported in Table 1.

### PCA

The analysis was carried out on a sample of over 1,000 respondents, a number beyond which test parameters tend to be stable regardless of the participant-to-variable ratio. The Kaiser–Meyer–Olkin sampling adequacy measure attained fairly high values (Kaiser–Meyer–Olkin value = 0.755), demonstrating that communalities were high and the sample's correlation matrix was appropriate for the analysis to proceed (Mundfrom et al., 2005). When the 7 items used to assess imagined reactions to sexual harassment were analyzed, two factors emerged. The first factor (refer to Table 2), labeled as nonassertive reactions, was composed of the following reactions, confirming the theoretical conceptualization: (a) speak about the abuse with family and/or friends, (b) speak about the abuse with colleagues, and (c) avoid the intimate situation with the abuser.

**Table 2:** Principal component analysis (PCA) of the reactions to sexual harassment.

	**Factor 1**	**Factor 2**
**Items**	**assertive reactions**	**non-assertive reactions**
Silence	−0.871	
Denounce the abuser to the Police	0.813	
Denounce the abuser to University organizations	0.789	
Report the abuse to University Professors	0.516	
Speak about the abuse with family and/or friends		0.865
Speak about the abuse with colleagues		0.846
Avoidance of intimate situations with the abuser		0.648
Eigenvalues	4.68	1.13
Explained Variance	47.69%	16.15%

The second factor (refer to Table 2), the assertive reactions, was composed of the following reactions: (a) break the silence about the hypothesized abuse (inverse of silence), (b) denounce the abuser to university organizations, (c) denounce the abuser to the police, and (d) report the abuse to university professors.

*MANOVA*. A 2 × 4 (sex × gender roles) MANOVA was conducted on the two dependent variables, namely, assertive and nonassertive reactions.

Inspection of the cell sizes for comparisons of sex by gender role revealed unequal cell sizes ranging from 83 to 240 participants (refer to Table 3).

**Table 3:** Cell sizes of gender roles by sex.

**Gender roles**	**Sex**		**Total**
	**Males**	**Females**	
Feminine	86	212	298
Masculine	158	145	303
Androgynous	169	228	397
Undifferentiated	180	237	417
Total	593	822	1,415

Following the procedures recommended by Tabachnick and Fidell (2007), cell sizes were reduced *via* random deletion to the maximum ratio of 1:1.5. A total of 83 female participants were randomly removed from the Feminine Gender Role category. *Post-hoc* comparisons revealed a similar pattern of effects between the full data set and the data with randomly deleted cases.

**Figure 1:**
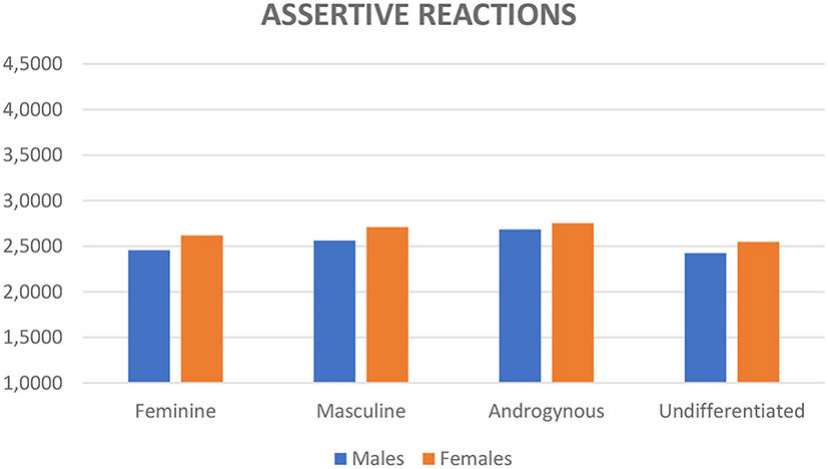
Mean scores of assertive reactions divided for sex and gender role according to the Bem (1974) theory.

**Figure 2:**
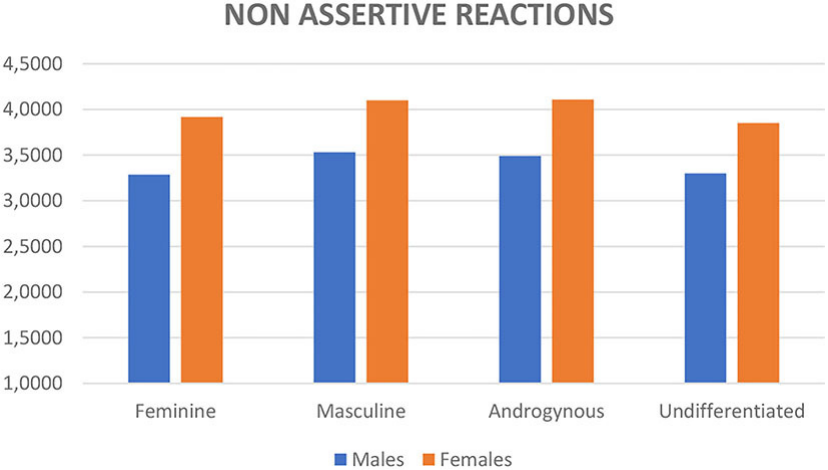
Mean scores of assertive reactions divided for sex and gender role according to the Bem (1974) theory.

The MANOVA revealed a significant main effect for sex [*F*_(2;1, 323)_ = 56.49, *p* < 0.001, Wilk's Λ = 0.92, partial η^2^ = 0.08] and gender role [*F*_(6;2, 646)_ = 3.42, *p* < 0.001, Wilk's Λ = 0.98, partial η^2^ = 0.01]. No significant interaction between sex and gender role was found. Mean scores are reported in Figure 1 for assertive reactions and in Figure 2 for nonassertive reactions.

*Post-hoc* comparisons using the Bonferroni correction on the four gender roles were conducted using the two separate measures of reactions to sexual harassment regarding assertive ones, the androgynous category reported significantly higher scores compared to feminine (*p* = 0.031) and undifferentiated (*p* = 0.002) and the masculine gender role was not significantly different from the other gender roles. When we considered the nonassertive reactions, the feminine category reported lower scores compared to the androgynous (*p* = 0.017) and the masculine (*p* = 0.004) gender role categories; moreover, the masculine category reported higher scores when compared to the undifferentiated category (*p* = 0.029); this latter category reported lower scores when compared to the androgynous category (*p* = 0.006). When we compared sex, women reported higher scores for both types of reactions (*F*_(1;1, 331)_ = 5.68, *p* < 0.05, partial η*p*^2^ = 0.004; *F*_(1;1, 331)_ = 101.33, *p* < 0.001, partial η*p*^2^ = 0.071, respectively.

## Discussion

There is a wide collection of evidence that SH is a persistent phenomenon in academia, occurring daily, is prevalent and widespread, and has devastating professional and personal effects on the targeted person (Mansfield et al., 2019). A 2018 US National Academies of Sciences, Engineering, and Medicine (NASEM) report documented the painful experiences and myriad effects of unwanted sexual attention, gender harassment, and sexual coercion. The report also emphasizes that women of color and gender nonconforming women face multiple, intersecting forms of harassment. In sum, the literature suggests that harassment is still present and pervasive in academia. However, the existing literature on SH and sexism in academia is limited in scope, and many studies have relatively small sample sizes. There is a need to better understand the SH experiences and patterns in academia (Seto, 2019), especially the coping mechanism adopted by the victims and the institutional responses to SH reporting. Responding to the harassment is a process and not a single act with an expiring date; there are numerous ways in which victims attempt to manage their situation, of which formal reporting is typically the last resort.

Our results showed that, contrary to our hypothesis (Rudman et al., 1995; Popoola, 2010; Arulogun, 2013), women react more than men in both assertive and nonassertive modalities. It is plausible that considering the SH phenomenon as predominantly targeting girls and adult women, those react more than their male peers in both ways, assertive and nonassertive, because they are called to face the problem more frequently compared to men with a significant potential burden of reacting in the appropriate way to stop the abuse. As reported in a seminal study by Fitzgerald et al. (1995, p. 118), “legal proceedings... in practice if not theory, hold the victim responsible for responding ‘appropriately',”... “placing the burden of non-consent on the victim.” The authors highlighted that, up to that point in time, frameworks for understanding women's responses to SH were typically based on an assumption that reactions were typically viewed as simply more or less assertive, placing all the responsibility on the victim. Recent literature argued that the answers and services provided by the universities, namely, organizational and institutional factors, play a key role in the complex and dynamic experience of reporting the SH in a context that potentially could operate secondary victimization. In an effort to better understand the SH experiences of women in science, technology, engineering, and mathematics (STEM) fields, the National Academies Committee on the Impacts of Sexual Harassment in Academia commissioned the Research Triangle Institute (RTI) to conduct a series of interviews (Lindquist and McKay, 2018). The results showed that women had numerous ways of coping with SH, e.g., adopting internal coping mechanisms, like minimizing or normalizing the incidents (e.g., trying to ignore or laugh it off); facing the harasser; engaging in mindfulness, spiritual, and self-healing activities or in exercise or physical activity; and staying focused on their careers (Lindquist and McKay, 2018). Women also reached out to friends and family, which was considered almost universally to be a positive choice. However, reactions from colleagues turned out to be a mixed bag for these women, encompassing supportive and emphatic answers and, at the same time, the second victimization: they were not believed or ridiculed. Results from the 2016 ARC3 survey at the University of Texas System confirm that students have very low reporting rates, with only 2.2% of all students who experienced SH reporting it to the institution and 3.2% disclosing the experience to someone in a position of authority at the institution. In a study on graduate students, only 6.4% of those who had been sexually harassed reported the incident (Rosenthal et al., 2016). As a coping mechanism, formal reporting for targets is the last resort: it becomes an option only when all others have been exhausted. Cortina and Berdahl (2008, p. 484) motivated the reluctance to use formal reporting by the “fear of blame, disbelief, inaction, retaliation, humiliation, ostracism, and damage to one's career and reputation.” These fears are justified because reporting processes often bring few benefits and many costs to the targets. In particular, students are often reluctant to start the formal reporting process with their campus because of fear of reprisals, retaliation, bad outcomes, not knowing adequately formal procedures, concerns about confidentiality issues, and fear that the institutional process will not serve them or even will damage them (Pappas, 2016). Recently, Hershcovis et al. (2021) in an attempt to explain the social dynamics of SH silencing (vs. reporting) of the violence affirmed that nonassertive reactions are safer in academia because universities' network compositions and belief systems serve to socially compel network silence, which enables SH to persist. Hershcovis et al. (2021) theorized the reactions to SH as a network phenomenon, introducing the concept of network silence around SH, defined as having three components, namely, being silent, silencing, and not hearing. The authors cast a wide net on the scope of responsibility for silence beyond victims to members of the social network, witnesses, and authority figures comprised, highlighting how silence is socially generated by examining the network elements that coerce and support silence. Using punishments, rewards, reinforcements, and mimicry, network members learn to not report, to silence each other, and to not hear when it comes to SH, making these three subcomponents of network silence form a mutually reinforcing pattern. The authors proposed potential explanations for the female students of our sample who showed higher scores in assertive reactions. Concerning the influence of the second individual variable considered, i.e., gender role, this has been more equivocal (Bursik and Gefter, 2011) with some researchers reporting significant interactions of gender and gender role in perceptions of SH (Powell, 1986; Russell and Trigg, 2004), while others report nonsignificant main and interaction effects for gender role on perceptions of SH in academic contexts (Bursik, 1992). Our results confirmed the main effect of both sex and gender roles on students' assertive and nonassertive reactions to SH in academia but their interaction was not confirmed such as in seminal research on the topic investigated (see Bursik, 1992). Although the perception of gender roles may play a subtle role, its impact on the perception of SH has not received extensive empirical support in recent times.

This study has some limitations: the study is conducted with a cross-sectional research design, which prevents the formulation of any causal inferences. Longitudinal or experimental studies testing the existence of causal relations are a challenge for future research. Research on informal and formal reporting mechanisms should be investigated further and the scales used to assess the reactions of SH have to be refined in future studies to capture more broadly the complex experience of abuse reporting, especially when the abuse comes from a person in a position of power over the victim.

## Conclusion, policy implications, and future directions

Sexual harassment is a diffused problem that tends to be underestimated in research centers and universities. However, recent analyses and reviews, undertaken among others in the context of European Union (EU)-funded structural-change projects, have revealed the pressing need for action against this problem (see European Institute for Gender Equality, 2016). Recent literature showed that organizational and institutional factors play a key role in the experience of reporting the abuse in a context like academia in which SH potentially has been evaluated as a norm and which could operate as secondary victimization. Educational programs about SH may prove useful in preventing its occurrence.

In the context of the EU, many educational programs were developed that proposed some useful guidelines and recommendations implemented to face SH with a focus on the institutional role (see European Institute for Gender Equality, 2016 for a list of guidelines developed in France, Spain, and the UK). In any case, it should be clear that abuses in any form, neither physically nor psychologically, are not tolerated. Gender equality plans in academia (Aru et al., 2020) can improve a nonsexist and safe environment for students. It is important to create diverse, inclusive, and respectful environments and move beyond legal compliance to address culture and climate. SH needs to be faced as a problematic issue that requires institutional leaders to engage with and listen to students and other campus community members. It is urgent to improve transparency and accountability of policies on the management of SH: academic institutions need to formulate a clear, easily accessible, and consistent procedure to facilitate SH reporting, considering the sensitive balance of confidentiality and transparency issues. It is also essential to provide support for the victims (e.g., social services, healthcare, legal representation, and advice concerning career development). Funders should support the following critical research areas: SH of women in underrepresented and/or vulnerable groups, including women of color, disabled women, immigrant women, and sexual- and gender-minority women. Mechanisms for protecting victims from retaliation should be developed alongside approaches for mitigating the negative impacts experienced. The social construction of SH needs to be expanded to include interactions that do not necessarily involve physical contact or assault. The inclusion of specific definitions and examples of both overt and more subtle forms of sexual harassment behavior may be necessary for men and women to identify this type of behavior as problematic.

## Data availability statement

The raw data supporting the conclusions of this article will be made available by the authors, without undue reservation.

## Ethics statement

The studies involving human participants were reviewed and approved by Ethical Committee of University of Cagliari Prot. No. 0000895. The patients/participants provided their written informed consent to participate in this study.

## Author contributions

Material preparation and data collection were performed by CCab, EC, CCas, and LR. Statistical analyses were performed by OM, CS, and MA. The first draft of the manuscript was written by CCab, CS, OM, and MA. All authors commented on previous versions of the manuscript. All authors contributed to the study's conception and design. All authors read and approved the final manuscript.

## Funding

This research has received funding from University of Cagliari, SUPERA Project, European Union's Horizon 2020 Research and Innovation Programme under Grant Agreement No. 787829.

## Conflict of interest

The authors declare that the research was conducted in the absence of any commercial or financial relationships that could be construed as a potential conflict of interest.

## Publisher's note

All claims expressed in this article are solely those of the authors and do not necessarily represent those of their affiliated organizations, or those of the publisher, the editors and the reviewers. Any product that may be evaluated in this article, or claim that may be made by its manufacturer, is not guaranteed or endorsed by the publisher.

## References

[B1] Aru M. Balia S. Barbieri B. Cabras C. Cadeddu G. Carboni P.. (2020). Piano di uguaglianza di genere dell'Università degli Studi di Cagliari. Cagliari: UniCa Press.

[B2] Arulogun S. (2013). Experience of sexual harassment and coping strategies among students of the school of nursing of a tertiary hospital in Southwest Nigeria. Int. J. Nurs. Midwifery 5, 70–75. 10.5897/IJNM2013.0099

[B3] Bem S. (1981). Gender schema theory: A cognitive account of sex typing. Psychol. Rev. 88, 354–364. 10.1037/0033-295X.88.4.354

[B4] Bem S. L. (1974). The measurement of psychological androgyny. J. Consult. Clin. Psychol. 42, 155–162. 10.1037/h0036215 4823550

[B5] Benson D. J. Thomson G. E. (1982). Sexual Harassment on a University Campus: the confluence of authority relations, sexual interest, and gender stratification. Soc. Prob. 29, 236–251. 10.2307/800157

[B6] Bursik K. (1992). Perceptions of sexual harassment in an academic context. Sex Roles 27, 401–412. 10.1007/BF00289948

[B7] Bursik K. Gefter J. (2011). Still stable after all these years: perceptions of sexual harassment in academic contexts. J. Soc. Psychol. 151, 331–349. 10.1080/00224541003628081 21675185

[B8] Cabras C. Marmillata S. Sechi C. (2018). Sexual objectification in education: how do teachers perceive and evaluate students? Soc. Psychol. Educ. 21, 743–757. 10.1007/s11218-018-9432-3

[B9] Cabras C. Marmillata S. Tumatis R. Sechi C. (2022). Do personal factors make women and men more susceptible to self-objectification and the development of dysfunctional eating attitudes? Curr. Psychol. 41, 924–934. 10.1007/s12144-020-00622-6

[B10] Cairns K. V. (1997). Femininity and women's silence in response to sexual harassment and coercion. In: Sexual Harassment: Contemporary Feminist Perspectives. p. 91–111.

[B11] Cantor D. Fisher B. Chibnall S. H. Townsend R. Lee H. Thomas G.. (2015). Report on the AAU campus climate survey on sexual assault and sexual misconduct. Westat 3129, i-A6-8.

[B12] Cochran C. C. Frazier P. A. Olson A. M. (1997). Predictors of Responses to Unwanted Sexual Attention. Psychol. Women Q. 21, 207–226. 10.1111/j.1471-6402.1997.tb00109.x31718407

[B13] Cortina L. M. Berdahl J. L. (2008). Sexual harassment in organizations: A decade of research in review. Handbook Organ. Behav. 1, 469–497. 10.4135/9781849200448.n26

[B14] Cortina L. M. Wasti S. A. (2005). Profiles in coping: responses to sexual harassment across persons, organizations, and cultures. J. Appl. Psychol. 90, 182. 10.1037/0021-9010.90.1.182 15641899

[B15] De Leo D. Villa A. Magni G. Andreatta A. Gagliardi A. (1985). Presentazione della versione italiana e contributo alla taratura del Bem Sex-Role Inventory. Bollettino Di Psicologia Applicata, 175, 21–28.

[B16] European Institute for Gender Equality EIGE. (2016). Gender equality in academia and research GEAR tool. Luxembourg: Publications Office of the European Union. ISBN 978-92-9493-637-0

[B17] Fasting K. Chroni S. Knorre N. (2014). The experiences of sexual harassment in sport and education among European female sports science students. Sport Educ. Soc. 19, 115–130. 10.1080/13573322.2012.660477

[B18] Fischer A. R. (2006). Women's Benevolent Sexism as Reaction to Hostility. Psychology of Women Quarterly, 30, 410–416. 10.1111/j.1471-6402.2006.00316.x18945919

[B19] Fitzgerald L. F. Cortina L. M. (2017). “Sexual harassment in work *organizations: A view from the twenty-first century*,” in APA Handbook of the Psychology of Women. APA.

[B20] Fitzgerald L. F. Shullman S. L. Bailey N. Richards M. Swecker J. Gold Y.. (1988). The incidence and dimensions of sexual harassment in academia and the workplace. J. Vocat. Behav. 32, 152–175. 10.1016/0001-8791(88)90012-7

[B21] Fitzgerald L. F. Swan S. Fischer K. (1995). Why didn't she just report him? The psychological and legal implications of women's responses to sexual harassment. J. Social Issues 51, 117–138. 10.1111/j.1540-4560.1995.tb01312.x

[B22] Gruber J. Bjorn L. (1986). Women's responses to sexual harassment: an analysis of sociocultural, organizational, and personal resource models. Soc. Sci. Q. 67, 814–826. https://search.proquest.com/docview/1291767122?pq-origsite=gscholarandfromopenview=trueandimgSeq=1

[B23] Gruber J. E. (1992). A typology of personal and environmental sexual harassment: research and policy implications for the 1990s. Sex Roles 26, 447–464. 10.1007/BF00289868

[B24] Gutek B. A. (1985). Sex and the Workplace. San Francisco, CA: Jossey-Bass.

[B25] Gutek B. A. Koss M. P. (1993). Changed women and changed organizations: consequences of and coping with sexual harassment. J. Vocat. Behav. 42, 28–48. 10.1006/jvbe.1993.100315911507

[B26] Helgeson V. S. (1994). Prototypes and dimensions of masculinity and femininity. Sex Roles 31, 653–682. 10.1007/BF01544286

[B27] Hershcovis M. S. Vranjes I. Berdahl J. L. Cortina L. M. (2021). See no evil, hear no evil, speak no evil: theorizing network silence around sexual harassment. J. Appl. Psychol. 106, 1834–1847. 10.1037/apl0000861 33600193

[B28] Hill C. Silva E. (2005). Drawing the Line: Sexual Harassment on Campus. American Association of University Women's Educational Foundation. Available online at: https://eric.ed.gov/?id=ED489850

[B29] Huerta M. Cortina L. M. Pang J. S. Torges C. M. Magley V. J. (2006). Sex and power in the academy: modeling sexual harassment in the lives of college women. Pers. Soc. Psychol. Bull. 32, 616–628. 10.1177/0146167205284281 16702155

[B30] Hulin C. L. Fitzgerald L. F. Drasgow F. (1996). “Organizational influences on sexual harassment,” in Sexual Harassment in the Workplace: Perspectives, Frontiers, and Response Strategies, ed M. S. Stockdale (Thousand Oaks, CA: Sage Publications, Inc.), 127–150. 10.4135/9781483327280.n7

[B31] Jussen L. Lagro-Janssen T. Leenders J. Logie C. Mijdam R. (2019). Underreported and unknown student harassment at the Faculty of Science. PloS ONE 14, e0215067. 10.1371/journal.pone.0215067 PMC6483172 31022214

[B32] Karami A. White C. N. Ford K. Swan S. Spinel M. Y. (2020). Unwanted advances in higher education: Uncovering sexual harassment experiences in academia with text mining. Inf. Process. Manage. 57, 102167. 10.1016/j.ipm.2019.102167

[B33] Knapp D. E. Faley R. H. Ekeberg S. E. Dubois C. L. (1997). Determinants of target responses to sexual harassment: a conceptual framework. Acad. Manage. Rev. 22, 687–729. 10.2307/259410

[B34] Leitich K. A. (1999). Sexual harassment in higher education. Education 119, 688–692.

[B35] Lindquist C. McKay T. (2018). Sexual Harassment Experiences and Consequences for Women Faculty in Science, Engineering, and Medicine. Research Triangle Park, NC: RTI Press. 31216159

[B36] Magley V. J. (2002). Coping with sexual harassment: Reconceptualizing women's resistance. J. Pers. Soc. Psychol. 83, 930–946. 10.1037/0022-3514.83.4.930 12374445

[B37] Magley V. Hulin C. Fitzgerald L. F. DeNardo M. (1999). Outcomes of self-labeling sexual harassment. J. Appl. Psychol. 84, 390–402. 10.1037/0021-9010.84.3.390 10380419

[B38] Mansfield B. Lave R. McSweeney K. Bonds A. Cockburn J. Domosh M.. (2019). It's time to recognize how men's careers benefit from sexually harassing women in academia. Human Geogr. 12, 82–87. 10.1177/194277861901200110

[B39] Matsui T. Kakuyama T. Onglatco M-. L. Ogutu M. (1995). Women′s perceptions of social-sexual behavior: a cross-cultural replication. J. Vocat. Behav. 46, 203–215. 10.1006/jvbe.1995.1013

[B40] McCabe M. P. Hardman L. (2005). Attitudes and perceptions of workers to sexual harassment. J. Soc. Psychol. 145, 719–740. 10.3200/SOCP.145.6.719-740 16334516

[B41] Mundfrom D. J. Shaw D. G. Ke T. L. (2005). Minimum Sample Size Recommendations for Conducting Factor Analyses. Int. J. Test. 5, 159–168. 10.1207/s15327574ijt0502_421521091

[B42] National Academies of Sciences Engineering Medicine. (2018). Sexual Harassment of Women: Climate, Culture, and Consequences in Academic Sciences, Engineering, and Medicine. Available online at: https://www.nap.edu/catalog/24994/sexual-harassment-of-women-climate-culture-and-consequences-in-academic (accessed September 1, 2021). 29894119

[B43] Nicolson P. (1997). “Gender inequality, sexual harassment and the toxic organization: the case of medical women,” in Sexual Harassment: Contemporary Feminist Perspectives. p. 32–48.

[B44] Pappas B. A. (2016). Dear Colleague: Title IX coordinators and inconsistent compliance with the laws governing campus sexual misconduct. Tulsa l. Rev., 52. 10.3366/ajicl.2011.0005

[B45] Pina A. Gannon T. A. Saunders B. (2009). An overview of the literature on sexual harassment: Perpetrator, theory, and treatment issues. Aggress. Violent Behav. 14, 126–138. 10.1016/j.avb.2009.01.002

[B46] Popoola B. (2010). Peer sexual harassment and coping mechanisms of female students in a Nigerian University. Edo J. Counsell. 1, 34–49. 10.4314/ejc.v1i1.52381

[B47] Powell G. N. Effects of sex role identity and sex on definitions of sexual harassment. Sex Roles 14, 9–19. (1986). 10.1007/BF00287844

[B48] Rosenthal M. N. Smidt A. M. Freyd J. J. (2016). Still second class: Sexual harassment of graduate students. Psychol. Women Q. 40, 364–377. 10.1177/0361684316644838

[B49] Rudman L. A. Borgida E. Robertson B. A. (1995). Suffering in silence: procedural justice versus gender socialization issues in university sexual harassment grievance procedures. Basic Appl. Soc. Psychol. 17, 519–541. 10.1207/s15324834basp1704_6

[B50] Russell B. L. Trigg K. Y. (2004). Tolerance of sexual harassment: an examination of gender differences, ambivalent sexism, social dominance, and gender roles. Sex Roles 50, 565–573. 10.1023/B:SERS.0000023075.32252.fd

[B51] Sabbag A. Garfield J. Zieffler A. (2018). Assessing statistical literacy and statistical reasoning: the real instrument. Stat. Educ. Res. J. 17, 141–160. 10.52041/serj.v17i2.163

[B52] Seto M. C. (2019). Advancing our scientific understanding of sexual harassment. Arch. Sex. Behav. 48, 1641–1643. 10.1007/s10508-019-1426-5 30805829

[B53] Stockdale M. Hulin C. L. Fitzgerald L. F. Drasgow F. (2014). “Organizational influences on sexual harassment,” in Sexual Harassment in the Workplace: Perspectives, Frontiers, and Response Strategies (Thousand Oaks, CA, SAGE Publications, Inc), 127–150.

[B54] Tabachnick B. G. Fidell L. S. (2007). Using Multivariate Statistics 5. Boston: Pearson Allyn and Bacon.

[B55] Taiwo M. O. Omole O. C. Omole O. E. (2014). Sexual harassment and psychological consequence among students in higher education institutions in Osun State, Nigeria. Int. J. Appl. Psychol. 4, 13–18. 10.5923/j.ijap.20140401.02

[B56] Thakur M. B. Paul P. (2017). Sexual Harassment in academic institutions: A conceptual review. J. Psychosoc. Res. 12, 33–40.

[B57] Willness C. R. Steel P. Lee K. (2007). A meta-analysis of the antecedents and consequences of workplace sexual harassment. Pers. Psychol. 60, 127–162. 10.1111/j.1744-6570.2007.00067.x

[B58] World Medical Association (2001). World Medical Association declaration of Helsinki: Ethical principles for medical research involving human subjects. Bull. World Health Organ. 79:373–4. 11357217 PMC2566407

