# Supplementary material for: To be assertive or not to be assertive: That is the question! Students' reactions to sexual harassment in academia
Source: Front Psychol. 2022 Sep 20;13:949103. doi: 10.3389/fpsyg.2022.949103 (PMC9530442; doi:10.3389/fpsyg.2022.949103)
Supplement: Supplementary file 1 [file Data_Sheet_1.docx]

Questionario sulle Reazioni alle Molestie Sessuali in Accademia (**QRMSA**) (Versione Italiana)

Il questionario intende con l’espressione “molestie sessuali” quei comportamenti indesiderati a connotazione sessuale, espressi in forma fisica, verbale o non verbale, aventi lo scopo o l’effetto di violare la dignità di una lavoratrice o di un lavoratore e di creare un clima intimidatorio, ostile, degradante, umiliante o offensivo” (Codice di Pari Opportunità, art. 26)^*^.

| **La sua reazione a fronte di una molestia subita nel suo ambiente di lavoro all’Università potrebbe essere:** | ***Mai*** | ***Raramente*** | ***Qualche volta*** | ***Spesso*** | ***Sempre*** |
| --- | --- | --- | --- | --- | --- |
| Il silenzio |  |  |  |  |  |
| Parlarne con familiari e/o amici/amiche |  |  |  |  |  |
| Parlarne con colleghe e colleghi |  |  |  |  |  |
| Parlarne con un professore/professoressa |  |  |  |  |  |
| Denunciare l‘accaduto alle figure istituzionali dell’ateneo |  |  |  |  |  |
| Sporgere denuncia alle forze dell’ordine |  |  |  |  |  |
| Evitare di incontrare quella persona da solo/a |  |  |  |  |  |

Reactions to Sexual Harassment in Academia Questionnaire **(RSHAQ) (**English Version)

In the following questionnaire, the expression "sexual harassment" refers to “those unwanted behaviors with sexual connotations, expressed in physical, verbal or non-verbal form, having the purpose or the effect of violating the dignity of a female or a male worker and creating an intimidating, hostile, degrading, humiliating or offensive climate" (Equal Opportunities Code, Art. 26)**.

| **Your reaction to being harassed in your work environment at the University could be:** | ***Never*** | ***Seldom*** | ***Sometimes*** | ***Often*** | ***Always*** |
| --- | --- | --- | --- | --- | --- |
| Silence |  |  |  |  |  |
| Speak about the abuse with family and/or friends |  |  |  |  |  |
| Speak about the abuse with colleagues |  |  |  |  |  |
| Report the abuse to University Professors |  |  |  |  |  |
| Denounce the abuser to University organizations |  |  |  |  |  |
| Denounce the abuser to the Police |  |  |  |  |  |
| Avoidance of intimate situations with the abuser |  |  |  |  |  |

*Codice delle Pari Opportunità (D.lgs. 11 aprile 2006, n. 198),  Art.26 - Molestie Sessuali

**Equal Opportunities Code (Legislative Decree no. 198/2006), Art.26 - Sexual Harassment
